# Editorial: Maximizing local government impact on community health initiatives

**DOI:** 10.3389/fpubh.2025.1660969

**Published:** 2025-08-04

**Authors:** Karolina Sobczyk, Marlena Robakowska

**Affiliations:** ^1^Department of Economics and Health Care Management, Faculty of Public Health in Bytom, Medical University of Silesia, Katowice, Poland; ^2^Department of Public Health and Social Medicine, Medical University of Gdansk, Gdansk, Poland

**Keywords:** local governments unit, public health, health program, health promotion, health economics

## Background

Local governments occupy a critical position in public health governance due to their influence over key social determinants of health, such as housing, transportation, education, land use, and environmental regulation. According to the theory of social determinants of health, these non-medical factors are among the most powerful influences on population health outcomes (1, 2). To address the complex and interrelated nature of health challenges, the “Health in All Policies” (HiAP) approach has gained prominence. HiAP is a governance strategy that integrates health considerations into policymaking across all sectors, not just the health sector. It rests on three main pillars: intersectoral collaboration, systems thinking, and a commitment to health equity (2, 3).

Intersectoral collaboration emphasizes the need for coordinated efforts among various municipal departments and external partners, recognizing that no single sector can address public health challenges alone. Systems thinking provides a framework for understanding how different policy areas interact and influence health over time. The focus on health equity underscores the moral and practical imperative to address avoidable disparities, particularly among marginalized or disadvantaged groups (1, 3). Theoretical models of local health governance—including HiAP, the theory of health equity, and community health frameworks—stress the importance of participatory governance. Community engagement in decision-making processes enhances the relevance, effectiveness, and sustainability of public health interventions, while also fostering trust and civic empowerment (1, 2).

Effective public health policy at the municipal level therefore involves more than delivering healthcare services. It requires strategic management of the social and environmental contexts in which people live. Data-informed policymaking is essential, enabling local authorities to target interventions where they are most needed, evaluate outcomes, and adjust strategies accordingly. Ultimately, the theoretical foundation of local public health governance points toward integrated, equity-driven, and participatory approaches. By embedding health into all aspects of local policy and planning, municipalities can act as powerful agents in creating conditions that support healthier, more resilient communities (1, 4).

This collection of 13 articles explores how local authorities can effectively address social determinants of health, reduce disparities, and implement sustainable, community-focused strategies. The contributions offer a wide range of perspectives, including empirical case studies, policy analyses, and conceptual frameworks. Across this diversity, common threads emerge: the importance of cross-sector collaboration, meaningful community engagement, and the use of data to inform action.

## Key contributions

- Khadka et al. examine barriers to implementing Nepal's national health policy at the local level. Lack of infrastructure, staff, and funding were major obstacles. Younger, tech-savvy workers were more effective implementers. The study advocates for training and better coordination.
- Li X. et al. study investment models in senior health in Japan and South Korea. Successful models combined fiscal investment, tech innovation, social capital, and institutional support. The authors highlight the need for flexible, context-specific public–private partnerships.
- Zhang et al. analyze how Wuhan's local government managed COVID-19 by allocating administrative attention across routine and emergency tasks. Their model helps explain decision-making under resource constraints but warns of attention fatigue.
- Jiang et al. assess how participatory budgeting in Chinese hospitals affects performance. While objective self-efficacy links were weak, perceived participation improved planning and communication, ultimately enhancing non-healthcare outcomes.
- Wang et al. review China's fragmented Health Impact Assessment (HIA) legislation. Despite pilot programs in 32 provinces, comprehensive regulation is lacking. The authors call for a unified HIA statute and capacity-building to integrate health into policy.
- Wei, Xu et al. compare three Chinese counties' responses to COVID-19. Counties with better insight, coordination, and learning capacities managed outbreaks more effectively. The study underscores the value of adaptive, responsive local governance.
- Zhu and Du explore public sports expenditure across China's provinces. Effective investment models integrated tech, cultural promotion, and housing support. Findings support cross-sector strategies to enhance physical activity participation.
- Stöllman et al. identify factors behind low sickness absence in Swedish municipalities: accessible leadership, continuous staff development, inclusive work environment management, and open communication. These practices promote organizational resilience.
- Lontano et al. present the CareVax protocol, integrating hospital and regional systems to improve vaccination among frail adults in Italy. Using secure data matching, the system identifies candidates for recommended vaccines and invites them to participate. The model could serve broader preventive care efforts.
- Wei, Wang et al. examine “pairing assistance” during China's COVID-19 crisis, where strong central coordination met local cooperation. A three-phase model—launch, decision, and implementation—illustrates how national and local governments worked together effectively.
- Peters et al. review tobacco control in the U.S., identifying uneven policy effects due to variations in design, overlapping regulations, and subgroup differences. They recommend more nuanced evaluations that account for equity and context.
- Xue et al. assess Traditional Chinese Medicine (TCM) in reducing antibiotic use among children. Herbal therapies and non-pharmaceutical methods showed promise, though challenges in standardization and policy integration remain.
- Li T. et al. explore consumer understanding of food recall notices in China. Despite concern for food safety, many struggled to interpret notices. Personalized, clear messaging increased trust and effectiveness.

## Summary

Several recurring challenges are identified—insufficient funding, limited staff capacity, political turnover, and fragmented authority structures. However, the articles also point to tangible opportunities: building skills and capacity within local governments; leveraging partnerships with community organizations to co-design and implement initiatives; using local data and evaluation to adapt strategies and build public and political support; and embedding health into all municipal policy areas, not only those traditionally linked to healthcare.

The message is clear: local governments are not peripheral actors in public health—they are essential to driving sustainable, equitable improvements in population wellbeing. When equipped with the right tools, partnerships, and leadership, municipalities can lead meaningful change.

As attention continues to shift upstream toward the social and environmental determinants of health, the role of local actors becomes increasingly central. Change does not always require national legislation or large-scale reform; it can begin with a city council decision, a school-based initiative, or a neighborhood-led program. Together, the articles in this collection provide a practical blueprint for how municipalities—regardless of size or resource level—can become true champions of health. Their insights offer valuable guidance for policymakers, researchers, and practitioners working to strengthen the health of communities from the ground up.
